# Lecture on the Diseases and Operations of the Teeth

**Published:** 1847-03

**Authors:** Edwin Saunders

**Affiliations:** Surgeon Dentist to St Thomas' Hospital, and to the London Institution for Diseases of the Teeth, &c. &c.


### ARTICLE VI.

*Lectures on the Diseases and Operations of the Teeth.*—Delivered at St. Thomas' Hospital, in the Session of 1844-5, by EDWIN SAUNDERS, Esq., M. R. C. S., Surgeon Dentist to St Thomas' Hospital, and to the London Institution for Diseases of the Teeth, &c. &c.

GENTLEMEN—From what has been already said of the treatment of cases of irregularity of the teeth, it will be evident that among the prime elements of the education of the dentist, a knowledge of mechanical principles, and of their various applications, stands pre-eminent. The pulley, as represented in the different forms of ligature, the wedge, as in the case described in the last lecture, the screw and the lever, are all, separately or together, enlisted in the cause of what may be, not inappropriately, termed dental mechanics. We have hitherto been considering those cases in which the two first-named mechanical powers are generally employed, in pursuing this subject we shall see that the last are not without their use.

Having pointed out the means of correcting that form of irregularity which consists of inclusion or recession, and which results from the upper tooth passing behind the lower row on closing the mouth, and consequently being founded within the dental arc, it now remains to consider the mode of reducing such teeth as are external to it. These cases, as has already been remarked, are of two kinds, those which result from mechanical causes, as the pressure of contiguous teeth, arising from an overcrowded state of the mouth, a want of expansion of the maxillary bones, or an irregular state of the opposite rows, and those which are altogether independent of these causes, and are due to a preternatural development of the upper jaw forwards, combined more or less with lateral contraction. In the former, the indications are of course to liberate such teeth by expanding the arch, or by removing one or more of the adjacent organs, according to the peculiarities of the case. It frequently happens that with inclusion of the lateral incisors, there occurs protrusion of the centrals, while the former are locked in by the cuspid teeth, which usually occupy a prominent position. If in these cases the teeth are not disproportioned to the maxillary bones, and especially if the denture be flattened at the sides, or opposite to the angles of the mouth, regularity may be effected without the sacrifice of any of the teeth. The simple eduction of the lateral incisors, so as to range between the centrals and cuspid teeth, will be required, and to accomplish this the more readily, small pieces of india rubber may be inserted between the teeth. This very simple but effective auxiliary in regulating, as well as in some other operations on the teeth, for the first employment of which I believe we are indebted to our trans-atlantic brethren, is, I fear not, held in due estimation amongst us. In cases in which the teeth are closely pressed together, and in which it is desirable, in order to facilitate future operations, that they should be liberated, the introduction of a piece of the caoutchouc stretched out and passed between the teeth, and after being allowed to collapse, cut off on both sides, will effect quickly, and without distress to the patient, all that could be desired. In cases in which the cuspid teeth have not become developed, but, in which the central are in advance of the lateral incisors, a simple ligature will be sufficient, applied as follows: The centre of the length of silk is to be passed in front of the central incisors, and being passed between these and the laterals, is to be carried behind the latter; the ends are then to be brought together in front of the centrals and firmly tied. It is obvious that such a ligature will act as a lever in pressing the central incisors inwards and everting the laterals.

In the lower row, on account of the little variation of size between the central and lateral incisors, any irregularity of ordinary occurrence may be obviated by the removal of one of either class. But in cases in which the incisors are of large size, and the bicuspids well developed, while the cuspid teeth are altogether external to the dental arc, the most facile mode of restoring regularity is the removal of the latter. An unfounded prejudice exists in the minds of many people, and of many medical practitioners, against the removal of this tooth in either row. Those, however, who have once performed this operation, or witnessed it, and have traced its after effects, will have no hesitation in resorting to it where such a course affords the only or most probable prospect of success. The bold form and prominent position of this tooth would not justify its extraction, except under very urgent circumstances, but where the incisor and bicuspid teeth are well arranged, and the cuspides are external to the denture; on account of the extreme difficulty of moving the latter, their extraction is imperatively demanded. It usually happens that the lateral incisors are thrown back, internal to the dental arch, so that on passing a ligature in front of the central incisors and behind the laterals, and first bicuspid teeth, the intervening space becomes shortly obliterated. From this care being for some time deferred, it is occasionally found that the removal of the lateral incisor is called for, as affording the best probable chance of eventual regularity; in such cases, if the points of the cuspid teeth be cut or filed off, an equally favorable result is obtained. We have here a case of a lady, in whose mother a similar deficiency is traceable, who has but two instead of four incisors in the lower jaw. Of this case I can speak with confidence, having had the opportunity of tracing the second dentition; although with respect to the mother, I entertained a certain amount of scepticism, knowing what strange mistakes have occurred in the hands of ill-informed practitioners. We have also on the table a natural jaw, in which the upper lateral incisors have been removed, and in which the character of the cuspid teeth has been so far modified, as to be with difficulty discernible, except on a close or suggested inspection.

With respect to such cases of irregularity of protrusion as arise from a crowded state of the denture, general rules can only be laid down, their particular application varying with the endless modifications which are presented in practice being necessarily left to the judgment and skill of the practitioner. Where it appears desirable to repress the dental circle, the first bicuspid teeth usually afford the readiest means of accomplishing the object; should, however, one of the lateral incisors exhibit symptoms of decay, or should the second bicuspid, or first molar, the latter by no means an unfrequent occurrence even at this age, be in a carious condition, its removal in either case might ultimately effect the object. Several circumstances, however, must be taken into consideration, as being necessary to the concurrence of this event. These are, first the age of the individual, and this condition will be still further modified by the temperament, average health, &c., and secondly, the relative position and direction of the irregular organ. Should the second molar tooth have become developed (and this you will remember occurs in the thirteenth year) the removal of the first permanent molar will produce but little and very tardy effect in arranging the teeth of the anterior part of the jaw. In a large number of cases, however, but little is required to effect regularity, and in such, this is all the interference that will be demanded. In others, such is the crowded state of the denture, that the abstraction of some tooth more immediately concerned in the production of irregularity is called for, and here the judgment is required to decide, in each particular case which tooth is most difficult of reduction, or the removal of which would soonest permit of eventual regularity. This will be in some a bicuspid, in others, a lateral incisor, in others again, a cuspid tooth, according to the position which it may occupy with respect to the neighboring organs. The circumstance which most urgently demands attention, is the direction of the axis of the tooth. If this, in the cuspid (usually the irregular tooth) should be backwards and downwards, the removal of the lateral, more especially if this should be a carious or imperfectly developed tooth, will be the eligible mode of treatment. If on the other hand, this tooth, should be developed obliquely forwards, or anterior to the lateral incisor, the extraction of some tooth posterior to it should be preferred.

We have hitherto confined our attention to cases of protrusion of one or more teeth arising from a crowded state of the denture; others, however, are occasionally encountered in practice in which this form of irregularity is produced or maintained independently of this cause. We have such an one represented in this diagram, in which one only of the central incisors is protruded, and which I have selected for illustration, because it exhibits, by contrast, in a very striking manner, both the deformity and the mode of treatment. In this case the left central incisor is developed obliquely forwards, involving by such malposition not only an unsightly protrusion of the upper lip, but a danger of fracture of the tooth, or contusion or laceration of the lip. The mode of treatment is as follows: A plate of gold or silver is adapted to the palate and gums as far as the bicuspid teeth on either side, to which it is firmly united by a ligature on each side. This mode of attachment, with that derived from its being fitted deeply in the palate, renders it a fixed point from which a pressure may be made in any desired direction upon the irregular tooth. This point being gained, the next consideration is the best mode of maintaining a *continued* traction, and thus accomplishing its reduction in the shortest possible period. An obvious and simple method of effecting this is to apply a ligature which has been passed through the plate in a convenient direction around the tooth. This, however, requires to be continually renewed, and it was, therefore, proposed to substitute for the silk a more elastic and continuously acting ligature. For this purpose caoutchouc was applied, but as this to a great extent loses its elastic power on receiving an increase of temperature, it was soon abandoned, and its place is supplied by an extremely ingenious application of the spiral spring. One end of a piece of tightly coiled gold wire is attached to some point near to the posterior margin of the plate while the other is fixed, in a state of tension, by means of a silk or wire ligature to the irregular tooth. Here then is an elastic force acting continuously, unaffected by variations of temperature, and without interference with the functions of mastication or deglutition. A convenient method of uniting the spring to the plate is to screw it on to a piece of wire bent at right angles, and soldered into the plate at some point in the direction in which it is desired to use traction upon the tooth. While its free end may be readily secured upon the irregular organ by turning down the two final rings so as to form a loop, through which a ligature or fine wire may be passed. To guard against or rather to provide for the puckering of the gum consequent upon the motion of the tooth, (which occurs more rapidly than absorption of the former structure,) it will be necessary to keep the anterior margin of the plate at this part free, or to cut it away altogether. As the tooth moves inwards and the tension of the spring becomes consequently diminished, its power may be again increased by shortening, by the excision of one or more coils. The supposed inconveniences of this mode of treatment, are the clogging of the spring by the debris of the food, &c., and the difficulty of regulating its tractive power. Of the first, I confess to having no injurious experience: but with respect to the second, it may be well to remind those who have not hitherto employed this very beautiful and ingenious method, that, on account of its being continuous, the power of the spring should be but slight. A most interesting case in which this method was employed, with the most flattering results, for the reduction of the whole of the incisors of a young lady, by Dr. Brewster of Paris, was reported in the French Lancet, and subsequently in the American Journal of Dental Science, pp. 258—261, vol. i. This case, obviously belongs more to the second class of deformities arising from preternatural development of the upper jaw, inducing a protrusion of the teeth in the anterior part of the denture. These are not only more difficult of reduction, but exhibit more or less a strong tendency to regain their old position, a tendency still further increased by the habit of closing the mouth with the upper incisors external to the lower lip. It is scarcely necessary to observe, that the reduction of such deformities becomes an object of great moment on account not only of detriment to the appearance, but of the difficulty of articulation, and the almost impossibility of employing the teeth as incisive or masticatory instruments.

It sometimes happens in these cases that the anterior teeth are so short and imperfectly developed, that, if brought into a regular position, their length would not be sufficient for due contact with the tongue in articulation. To effect this object, it has been proposed by Mr. George, the accomplished lecturer on dental surgery at University College, whose ingenuity is only excelled by his readiness to impart his discoveries and attainments to his professional brethren, to place the spiral springs for the traction inward, upon elastic bands of gold, which will have a tendency to urge the tooth downwards, and thus promote its development in this direction. Of the success of this plan in practice, I have had no experience, but the idea is sufficiently novel and ingenious to merit a notice in this history of the treatment of such cases.

In cases of abnormal development of the maxillary bones, and in which it is required to reduce several or the whole of the anterior teeth, I have found it desirable *in practice* to vary the method of treatment. Commencing with the plate adapted to the internal gum and palate with its elastic springs, I afterwards resort to external pressure; on the occurrence of any considerable congestion and puckering of the gum internal to the moving teeth. Thus the teeth having now been moved inwards, upon the tractive principle, to a certain extent, I adapt to their altered position a plate of elastic gold on their anterior or external surfaces. Having secured this in its position by ligatures, and prevented its being pressed up towards the gums, by fittings of gold, which rest on the cutting edge of the incisors; I now insert compressed wedges of wood, as before described, between the gold band and the teeth to be moved. In this way time is gained, by continuing the movement inwards without compressing, and thus increasing the irritation of the gums within, or posterior to the teeth. It has been recommended to employ the screw in the place of the wedge, and inasmuch as it affords the operator the means of applying an uniform and nicely graduated pressure, it certainly offers some advantages. It has always appeared to me, however, that the inelastic nature of the force, together with the irritation of the mucous membrane lining the lip, produced by the head of the screw, are objections which must be fatal to its general use. A curious specimen of German ingenuity in the possession of Mr. Lemale of Chandos street, exhibits the following mechanical arrangement. A strong bar is adapted to the external surface of the teeth of the superior denture, having its attachment to the molars. The lateral incisor on the left side is within the dental arch, while the right central is developed prominently forwards. Opposite to the latter is a screw, which may be turned by a small key; and for the eduction of the lateral is a complete windlass, to the roller of which the two ends of a ligature are secured, which has passed behind the irregular tooth. In all cases in which it is required to bring the tooth inwards, one practical obstacle presents itself in the swelling and puckering of the gum, arising from the absorption taking place more slowly in the latter structure than in the alveoli of the maxillary bones. To obviate the difficulty arising from this cause, it is desirable to leave the edge of the plate free posterior to the teeth about to be moved, and to cut away or raise it, from time to time, as may be found necessary. Notwithstanding these precautions, however, the gums may become so soft and irritable, and in such a state of congestion as to make it desirable to discontinue, for a brief interval, the use of the plate. Under these circumstances the simple bar of gold external to the teeth secured to the molars and made to exert a pressure by means of wooden wedges upon the irregular teeth, is a valuable and efficient substitute.

When the teeth present themselves in a transverse position constituting what may be termed torsion, a plate similar to that used in the last case, with fittings adapted to make pressure against the internal angle of the tooth, combined with a counteracting pressure on the opposite angle, may be employed. The treatment of these cases, at all times tedious and difficult, is needlessly prolonged from omitting to establish a decided counteracting pressure. A plate of gold attached to the bicuspid teeth, and prolonged against the internal angles of the irregular teeth forms a point d'appui, by means of which a counter pressure may be exerted on the external or everted angles. This is accomplished in the following simple manner; a hole is drilled through the plate opposite to the division between the incisors, through this a strong ligature is passed, which is firmly tied to an elastic gold bar, extending on either side for rather more than the entire width of the teeth. The ligature may be renewed every second or third day, according to its strength, or with reference to the temperament, health, and constitutional power of the patient, considerations which will never be absent from the mind of the careful and enlightened practitioner. When the tension is partial, and a less complicated apparatus is desired, a band of gold may be placed on both the external and internal surfaces of the irregular teeth, and firmly tied together by a ligature passing between them; but to prevent that on the internal surface from slipping down, a small piece of gold must be soldered at right angles to the bar extending up to the neck of each tooth, having a slight curve at the end to retain a ligature, by which it is to be fastened around the teeth. This will prevent the bars from becoming displaced, and will not interfere with the eversion of the teeth. Another mode by which this is effected, is shown in the diagram already referred to. A strong fitting of gold is to be securely adapted to the irregular tooth, and to this is to be united an elastic bar, in such a position as that when its free end is unattached, it will fly into the position indicated by the dotted or interrupted line. This, which constitutes in fact an elastic lever, is now to be secured by strong ligatures to one or more of the molar or bicuspid teeth, when its action is obviously a continued twisting of the irregular organ into the desired position. Occasionally the spiral spring may be with advantage substituted for the elastic lever, more especially if it be desired at the same time to effect any alteration in the relative position of the tooth. Much may be accomplished in the less strongly marked cases, by the daily application of a pressure in the desired direction, by means of a piece of wood deeply notched to receive the edge of the tooth. The forcible dislocation of the tooth, however, by a pair of forceps, as was formerly practised, and afterwards securing it in its position by ligatures ; of which practice rupture or strangulation of the cord of vessels entering the end of the root, was an almost inevitable consequence, cannot be too strongly reprobated. With respect to the treatment of irregularity of the teeth : What is done slowly is alone done surely; and, what is accomplished in this way would be scarcely believed by those who are strangers to the improved condition and ample resources of the dental art.

To attempt to describe minutely all the varieties of irregularity which are encountered in practice, as well as the modifications of apparatus which are required for their reduction, would be utterly futile ; the main features and broad principles have, however, been placed before you, their particular applications being left to individual acumen and skill. With the means already described, viz. the lever, the inclined plane, the pulley, the wedge, and the screw, we may, by a skilful application, and use of one or more of these powers, accomplish much in the way of correcting deformities, while by a judicious care and attention during the process of the second dentition, much more may be done towards preventing their occurrence. The causes of irregularity have been the subject of much comment, and of diverse opinion, some practitioners attributing its occurence to neglect in ridding the mouth at a sufficiently early period, of the temporary teeth; while others, asserting the competency of nature to fulfil her own functions, unaided by the interference of art, trace all mischief of this kind to the premature removal of the first set. Mr. Bell, in an honest zeal for the integrity of his profession, protests in unequivocal terms, against the practice of indiscriminate extraction of the temporary teeth, but it may perhaps be doubted, whether his invectives have not conducted to the opposite extreme. The safe practice is here, as in other cases, in the middle course, that is to say, if by the retention of the temporary teeth, even of those which are not yet destined in due course to be removed, an obstacle is opposed to the regular arrangement of the permanent organs, their extraction is not only justifiable, but imperative. On the other hand, to remove the teeth of the first set in anticipation, when no such reason exists, is not only to preclude eventual regularity, but to perpetrate a gross injustice in the form of a gratuitous infliction of suffering. In the examination of the mouth for this purpose, it will not suffice to confine the attention to the row in which the irregularity may occur, but the mouth must be closed in a variety of positions, for it sometimes happens that the retention of a temporary tooth in one row will produce irregularity in the permanent tooth of its opposite. This is frequently the case with the superior lateral incisor, with reference to the lower cuspid. From the almost infinite variety of cases, their complexities of character, and the gradations of deformity which are encountered, it becomes impossible to point out anything more than a broad and general line of practice; much, in individual cases, being necessarily left to the judgment and integrity of the practitioner.

In concluding this subject, we have only time to glance at those forms of irregularity which result from redundant and abnormal, commonly called supernumerary, teeth. These are almost invariably confined to the upper row, and occur between or behind the central incisors, sometimes occupying their place, at others, situated at the posterior and lateral parts of the jaw, as between the second and third molar teeth in the external angle. They are sometimes simply of a conical form, having a tortuous root, at others possessing somewhat the appearance of a molar tooth. Occasionally they are found united to the tooth near to which they are placed, at others they exhibit a totally independent root and separate articulation. The temporary tooth is also sometimes retained in its position for many years after its period of shedding has become due; and as in these cases the permanent tooth may be assumed as having suffered arrest of development, it is worth while to pause before removing the former, in order to ascertain what may be the probability of the further growth of the latter. In some cases of retarded dentition, even at a very advanced period, I have found pressure, as produced by wearing an artificial tooth, impart the needed stimulus to the arrested organ, it being in a few months equal in appearance and utility to the well developed tooth. Supernumerary teeth, as they are redundant and abnormal productions, may, almost in every case, be with advantage removed. To this, however, there are exceptions, for we have here a preparation in which a supernumerary tooth is developed between the central incisors, without causing irregularity, and the removal of which, as it would leave a vacuity in the centre of the upper denture, would be injudicious. There are also some other forms of abnormal arrangement not conducing to general irregularity, such is this in which the cuspid tooth is placed posterior to the first bicuspis; here again is a cast of a mouth in which there are five incisors in the upper row, three laterals, and two centrals, but without interfering with the regularity of the dental arc. Cases of this kind also are not without interest, in which a spontaneous and original separation or interspace occurs between the central incisors. The attempt to close this space is vain, for it will continually re-appear, and without any adequate apparent cause. This case, however, must not be confounded with that which will be hereinafter described as due to the loss of the molar teeth, the sustaining instruments of the jaw, and in which such separation occurs from the unchecked pressure of the lower row, and is readily distinguished by the obliquity of direction, as well as more or less looseness of the teeth.

